# Association of maternal high-risk fertility behavior and under-five mortality in Ethiopia: Community-based survey

**DOI:** 10.1371/journal.pone.0267802

**Published:** 2022-05-06

**Authors:** Melash Belachew Asresie, Gizachew Worku Dagnew

**Affiliations:** Department of Reproductive Health and Population Studies, School of Public Health, College of Medicine and Health Sciences, Bahir Dar University, Bahir Dar, Ethiopia; Sabzevar University of Medical Sciences, ISLAMIC REPUBLIC OF IRAN

## Abstract

**Background:**

High-risk fertility behavior is a major public health concern in low and middle-income countries including Ethiopia. Some studies show that the relationship between high-risk fertility behavior and child mortality has analyzed each fertility behavior individually. Yet, there are limited studies that have analyzed outcomes associated with the joint impact of high-risk fertility behaviors. Therefore, the objective of this study was to examine the individual and combined influence of high-risk fertility behavior on under-five mortalities in Ethiopia.

**Methods:**

Data from the 2016 Ethiopian Demographic and Health Survey were used analyzed. A total of 10,773 mothers who gave live births were included in the final analysis. Both descriptive and bivariate and multivariate logistic regression analyses were performed using STATA V.14.

**Results:**

Overall, 62.1%, 24.0%, and 2.3% of women experienced at least one, two, and three high-risk fertility behaviors, respectively. In the multivariable analysis, under-five mortality was significantly associated with a combination of two or more maternal high-risk fertility behaviors. The odds of under-five mortality among children of women who were engaged in a combination of two high-risk fertility behaviors (AOR = 2.17, 95%CI: 1.52–3.08) and three high-risk fertility behaviors (AOR = 3.69, 95%CI:1.80, 7.55) was higher compared to children of women who have not engaged any high-risk fertility behaviors.

**Conclusion:**

This study revealed that a single high-risk fertility behavior was not associated with under-five mortality, yet the presence of two or more maternal high-risk fertility behaviors was an important factor that increased the likelihood of under-five child death. Thus, special emphasis should be given to children of women who engage in a combination of high-risk fertility behaviors. Furthermore, more emphasis should be placed on increasing access to family planning services and raising awareness about high-risk reproductive behaviors among Ethiopian women.

## Background

Despite substantial improvements to child health outcomes over the past few decades, around 5.4 million children younger than 5 years still die every year globally, which is 39 deaths per 1000 live births [1]. Of which about 80% of under-five deaths occur in Sub-Saharan Africa and South Asia [1].

The Sustainable Development Goal (SDG) focuses to lessen under-five mortality rates to below 25 under-five death per 1000 live births at the end of 2030 [2]. Except if the present circumstance improves will fail to achieve SDG3 which aims to end preventable newborn deaths by 2030 [3, 4]. Even though global under-five mortality rates have declined, Sub-Saharan Africa keeps on continuing to be the region with the highest under-five mortality rates in the world, which was 76 deaths per 1000 live births in 2017, shared half of the world’s under-five deaths. It was 30% in 1990 [1].

Although various measures have been taken to decrease under-five mortalities most Sub-Saharan countries show very high under-five mortality [5]. All the more as of late, the Health sector transformation plan of Ethiopia has had a great impact on the reduction of under-five mortality in Ethiopia [6, 7]. Nevertheless, the under-five mortality rate in the nation is high, 67 under-five children died per 1000 live birth in 2016 [8]. The growth and transformation plan of Ethiopia has started to make plans for reducing under-five mortality rates to below 30 deaths per 1000 live births by 2035 [7]. Arriving at this low level will require improvements in the socio-economic status of the population as well as improvement in the direct services provided by the health sectors [9].

Previous studies have recognized several socio-demographic, economical, and nutritional risk factors of under-five mortality [10–13]. Besides, lack of skilled human resources, inadequate infrastructure, and low investment in the health system have significantly increased the rate of childhood mortality in low and middle-income coutries [14]. Maternal High-Risk Fertility Behaviors; too early or too late childbearing age, narrow birth interval, and too many births is one of the biodemographic risk factors, that have been linked to negative health outcomes for the child’s health [15, 16]. Evidence suggested that high-risk fertility behavior is widespread and has a significant cause of neonatal and under-five mortality in low- and middle-income countries [17–19]. A systematic review and meta-analysis study in East Africa showed that 58% of women had at least on risk fertility behavior [20]. In Ethiopia, more than three-fourths (77%) of reproductive-age women had at least one high-risk fertility behavior [21]. Another study in Ethiopia also showed that about 88% of women were engaged to at least one high-risk fertility behavior, 70.6% of women were exposed to high birth order(>3), followed by 56.6% and 15.2% of women engaged too old (>34 years at birth) and closed birth interval (<24months), respectively [18].

Numerous studies that showed the relationship between high-risk fertility behavior and child mortality have analyzed each fertility behavior individually. However, many women had two or more high-risk fertility behaviors in Ethiopia, about 45% of women had multiple high-risk fertility behaviors [21]. A few studies analyzed outcomes associated with the combined influence of maternal high-risk fertility behaviors [19]. Yet, such studies are limited in the Ethiopian context. Since, it is using a nationally representative sample, identifying such relationships will be paramount important in the development of effective prevention programs requires a better understanding of the individual and combined influence of maternal high-risk fertility behavior on child mortalities to achieve MDG 3. Thus, this study was aimed to examine the association between maternal high-risk fertility behavior and under-five mortalities with hypothesized that children from mothers with two or more maternal high-risk fertility behaviors would be more like to die.

## Materials and methods

### Data source

#### Study setting, study period, and study design

Data from the 2016 Ethiopian Demographic and Health Survey (EDHS) was used to analyze [8]. Ethiopia is administratively structured into nine regional states and two city administrations. EDHS was a cross-sectional community-based survey conducted from January 18, 2016, to June 27, 2016. The survey was implemented by the Central Statistical Agency at the request of the Federal Minister of Health [8].

#### Sample size and sampling technique

EDHS used a two-stage stratified cluster sampling technique to ensure representativeness at the national and regional levels. Initially, each region was stratified into urban and rural areas yielding 21 sampling strata. After stratification, a total of 645 enumeration areas (202 in urban areas and 443 in rural areas) were selected with probability proportional to enumeration area size based on the 2007 Ethiopia population and housing census. A household listing operation was carried out in all the selected enumeration areas from September to December 2015. Then, 28 households from each cluster were selected using a systematic random sampling technique from the household listening [8]. During the survey, a total of 16,650 households were interviewed from 18, 008 selected representative households. For individual interviews, 16,583 eligible women were identified from the interviewed households, and interviews with 15, 683 women aged 15–49 were completed [8]. Mothers were asked to provide information about their births and child survival within the five years preceding the survey. In this survey, 11, 022 women were given births during the five years preceding the survey. Of which 253 were excluded due to incomplete information. Thus, for this study 10,773 mother-child pairs were included.

### Study variables

#### Outcome of interest: Under-five mortality

The outcome variable for this study was the death of under-five children which is a dichotomous variable coded as “0” if the child is alive and “1” if the child is died before reaching the fifth birthday.

#### Exposure variable: Maternal high-risk fertility behavior

The exposure variable for this study was maternal high-risk fertility behavior, which was defined by EDHS and constructed using four variables; (i) mother aged <18 years at the time of birth, (ii) mother age >34 years at the time of birth;((iii) mother of a child born after a short birth interval (<24 months); and (iv)mother of high parity (>3 children) [8].

In this study, we coded the maternal high-risk fertility as (I) no any risk fertility behavior, (II) mother age <18 years; (III) mother age > 34 years); (IV) birth interval <24 months); (V) mother of high parity (>3 children)); (VI) any double high risk fertility behaviors (*mother age <18 years & birth interval <24 months or mother age > 34 years & birth interval <24 months or mother age > 34 years & births order >3 or births order >3 & birth interval <24 months)*, and (VII) Triple high-risk fertility behavior (*mother age > 34 years & birth interval <24 months & births order >3*).

We recoded the four double high-risk fertility behaviors (mother age <18 years & birth interval <24 months or mother age > 34 years & birth interval <24 months or mother age > 34 years & births order >3 or births order >3 & birth interval <24 months) into one named as “any double” because of some categories are too small for statistical analysis.

#### Covariates

Variables such as current age of women (15–34, 35–49), number of household members (≤4, ≥5), religion (non-Muslim, Muslim), residence (urban, rural), women’s educational status (no education, primary, secondary or above) women’s occupation (not working, working), wealth index (poor, middle, rich), marital status (single, married), type of toilet (unimproved, improved), type of water source(unprotected, protected), sex of household head (male, female), covered by health insurance (no, yes), face health care access problem (no, yes), pregnancy intention (want then, want later, wanted never), type of birth (single, multiple), place of delivery (home, health facility), sex of the baby (male, female), apply any things on the umbilical cord after delivery (no, yes), and perceived size of baby) were included as covariates. The poor wealth index category was created by merging poorer and poorest and the variable rich was constructed by merging richer and richest.

### Statistical analysis

Since the sampling of designing of EDHS was complex sample weight (weighted frequency and weighted percentage) was used for all analyses to give accurate estimates for the population parameters using STATA software version 14.1. Frequency distribution and descriptive statistics (proportion, mean, and standard deviation (SD)) were used to describe the characteristics of the study participants. A Chi-square analysis was done to describe the relationship between the independent variables and the dependent variable. Bivariate and multivariate logistic regression models were performed to examine the independent association of high-risk fertility behavior on under-five mortality. Covariates were entered simultaneously into all of the regression models These variables which were significant in bivariate setup (p<0.20) were included in the multivariate logistic regression. A 95% confidence interval was used to declare statistical significance. Multi-collinearity was checked before multivariate logistic regression analysis was done. The goodness of fit of the final model was tested by Hosmer-Lemeshow and the p-value was greater than 0.05.

### Ethical considerations

This analysis is based on secondary data obtained with a request from the Demographic and Health Survey Program online repository. The primary data were gathered in line with the national Ethical procedures.

## Results

### Characteristics of the sample

A total of 10,773 women were eligible for this study. Of these, 53% were aged 25–34 years and 55.6% of women were had no work. The mean age of women was 29.57+0.06 years. The majority, (89.1%) of women were from rural residents and 66.3% of women had no education. One-fourth (25.8%) of women were giving birth at health facilities and 86.4% of the household head were male. From the total sampled population, 46.9% of women belonged to the poor wealth index and only 10.2% had improved toilets (Table 1).

**Table 1:** Characteristics of the study participants by under-five mortality (n = 10,773), EDHS 2016.

Variables		Overall	Under-five mortality		Chi2 (p-value)
			No	Yes	
The current age of women					4.66(0.40)
	15–24	2, 377 (22.1)	2,254 (94.8)	123 (5.2)	
	25–34	5,713 (53.0)	5,418 (94.8)	295 (5.2)	
	35–49	2,683 (25.0)	2,515 (93.7)	168 (6.3)	
Education					4.52 (0.41)
	No education	7,147 (66.3)	6,735 (94.2)	412 (5.8)	
	Primary	2,875 (26.7)	2,734 (95.1)	141 (4.9)	
	Secondary or high	751 (7.0)	718 (95.6)	33 (4.4)	
Occupation					0.01 (0.99)
	No	5,994 (55.6)	5,668 (94.6)	26 (5.4)	
	Yes	4,779 (44.4)	4,519 (94 .6)	260 (5.4)	
Marital status					1.25 (0.45)
	Single	537 (5.0)	502 (93.5)	35 (6.5)	
	Married	10,236 (95.0(	9,684 (94.6)	552 (5.4)	
Religion					4.02 (0.23)
	Non-Muslim	6,304 (58.5)	5,985 (94.9)	319 (5.1)	
	Muslim	4,469 (41.5)	4,202 (94.0)	267 (6.0)	
Residence					4.78(0.24)
	Urban	1,175 (10.9)	1,127 (96.0)	48 (4.0)	
	Rural	9,598 (89.1)	9,060 (94.4)	538 (5.6)	
No- of a household member					33.53 (<0.01)
	< = 4	2,888 (26.8)	2,669 (92.4)	219 (7.6)	
	> = 5	7, 885 (73.2)	7,518 (95.3)	367 (4.7)	
Wealth index					2.57 (0.05)
	Poor	5,049 (46.9)	4,788 (94.8)	261 (5.2)	
	Middle	2,239 (20.8)	2,122 (94.7)	117 (5.3)	
	Rich	3,485 (32.3)	3,278 (94.0)	207 (6.0)	
Type of toilet					14.27 (<0.01)
	Not improved	9,672 (89.8)	9,118 (94.3)	554 (5.7)	
	Improved	1,101 (10.2)	1.069 (97.1)	32 (2.9)	
Type of water source					1.41 (0.49)
	Unprotected	4,718 (43.8)	4,475 (94.9)	243 (5.1)	
	Protected	6,055 (56.2)	5,712 (94.3)	344 (5.7)	
Sex of the household head					0.12 (0.94)
	male	9,309 (86.4)	8,803 (94.6)	506 (5.4)	
	female	1,464 (13.6)	1,384 (94.5)	80 (5.5	
Covered by Health insurance					7.54 (0.05)
	No	10,392 (96.5)	9,815 (94.4)	577 (5.6)	
	Yes	381 (3.5)	372 (97.8)	9 (2.2)	
Face health care access problem					1.54(0.43)
	No	6,532 (60.6)	6,191 (94.8)	341 (5.0)	
	Yes	4,241 (39.4)	3,996 (94.2)	245 (5.8)	
Pregnancy intention					8.62 (0.04)
	Wanted then	7,759 (72.0)	7,319 (94.3)	440 (5.7)	
	Wanted later	1,988 (18.5)	1,906 (95.9)	82 (4.1)	
	Unwanted	1,026 (9.5)	962 (93.7)	64 (6.3)	
Type of birth					113.84 (<0.01)
	Single	10,490 (97.4)	9960 (95.0)	530 (5.0)	
	Multiple	282 (2.6)	226 (80.0)	56 (20.0)	
Type of delivery					1.77(0.46)
	Normal	10,570 (98.1)	9,999 (94.6)	571 (5.4(	
	c/s	203 (1.9)	188 (92.4	15 (7.6)	
Place of delivery					11.81 (0.03)
	Home	7,989 (74.2)	7, 518 (94.1)	471 (5.9)	
	Health facility	2,784 (25.8)	2,669 (95.9)	115 (4.1)	
Sex of baby					28.88 (<0.01)
	Male	5,616 (52.1)	5,246 (93.4)	370 (6.6)	
	Female	5,157 (47.9)	4,941 (95.8)	216 (4.2)	
Apply anything on the umbilical cord after delivery					10.38 (0.05)
	No	8,874 (82.4)	8,361 (94.2)	513 (5.8)	
	Yes	1,899 (17.6)	1,826 (96.1)	73 (3.9)	
Perceived size of baby					7.13 (0.04)
	Small	3,450 (32.0)	3,248 (94.1)	202 (5.9)	
	Medium	4,492 (41.7)	4,279 (95.3)	213 (4.7)	
	Large	2, 831 (26.3)	2,660 (94.0)	171 (6.0)	
Types of a high-risk fertility behavior					42.54(0.01)	
	No any risk	4,082 (37.9)	3,883 (95.1)	199 (4.9)		
	Mother age <18 years	473 (4.4)	442 (93.6)	31(6.4)		
	Mother age > 34 years	78 (0.7)	74 (94.4)	4 (5.6)		
	Birth interval <24 months	588 (5.5)	549 (93.4)	39 (6.6)		
	Births order >3	2,959 (27.5)	2,837 (95.9)	122 (4.2)		
	Double risks	2, 344 (21.7)	2,182 (93.1)	162 (6.9)		
	Triple risks	249 (2.3)	220 (88.1)	219 (11.9)		

According to this studied sample, 62.1% (95%CI: 61.9–62.3) of women experienced at least one high-risk fertility behavior. The most common single high-risk fertility behavior was high parity (birth order>3) (27.5%), followed by a closed birth interval (5.5%). Two thousand and three hundred forty-four (21.7%) women had a combination of two high-risk fertility behaviors (95%CI: 21.4–21.6). The most common combination of two high-risk fertility behavior was maternal age at delivery (>34 years) and birth order (>3) (11.8%), followed by a birth interval (<24 months) and birth order (>3) (9.55). The combination of maternal age at delivery (<18 years) and closed birth interval (<24 months) was 0.4% and only one woman was had the combination of maternal age at birth (>34 years) and closed birth interval. Of the total study participants, 2.3% (95%CI: 2.2–2.4) of women experienced the combination of three high-risk fertility behaviors (Table 1).

Across the studied sample, 586 (5.4%) of children born within the past five years preceding the survey died before they celebrate their fifth birthday. One in five (20%) of twin children died before they reached their five years. As Table 1 shows in the chi-square analysis; the number of household members, type of toilet, wealth index, pregnancy intention, pregnancy type, place of delivery, covered by health insurance, sex of baby, apply anything on the cord after delivery, the perceived size of the baby and high-risk fertility behavior was associated with under-five mortalities at p-value <0.05.

### Bivariate and multivariate analysis

Variables mentioned in Table 2 were significantly associated with under-five mortality in the bivariate analysis at p-value <0.25. Yet, in the multivariate analysis only; type of birth, sex of baby, place of delivery, number of household members, type of toilet, and type of high-risk fertility behavior were significantly associated with under-five mortality at p-value <0.05. Children who were born at health facilities were 32% less likely to die before they rich their fifth birthday as compared to children born at home (AOR = 0.68;95%CI: 0.46–0.99). Besides, children who were female were 38% less likely to under-five death as compared to male children (AOR = 0.62; 95%CI:0.48,0.81). The odds of under-five mortality among children of women who were engaged in a combination of two high-risk fertility behaviors were 2.17 times higher compared to children of women who have not engaged in any high-risk fertility behavior (AOR = 2.17, 95%CI: 1.52–3.08). Children of women who were engaged in the combination of three high-risk fertility behaviors had 3.69 times higher odds of death (AOR = 3.69, 95%CI:1.80, 7.55) as compared to children of women who have not engaged in any high-risk fertility behaviors (Table 2).

**Table 2:** Bivariate and multivariate association between high-risk fertility behaviors in women aged 15–49, other characteristics, and under-five mortality, EDHS 2016.

Variables		Crude Odds Ratio (COR)	Adjusted Odds Ratio (AOR)
No- of a household member			
	< = 4	1	1
	> = 5	0.60 (0.45, 0.79)	0.38 (0.27, 0.54)***
Wealth index			
	poor	1	1
	Middle	1.02 (0.72, 1.45)	1.10(0.76, 1.57)
	Rich	1.16 (0.86, 1.58)	1.51 (1.10, 2.07) *
Type of toilet			
	Not improved	1	1
	Improved	0.50 (0.30, 0.83)	0.49 (0.30, 0.81)**
Covered by Health insurance			
	No	1	1
	Yes	0.39 (0.12, 1.20)	0.42 (0.13, 1.31)
Pregnancy intention			
	Wanted then	1	1
	Wanted later	0.71 (0.49, 1.03)	0.76 (0.51, 1.12)
	Unwanted	1.11 (0.71,1.72)	1.00(0.62, 1.49)
Type of birth			
	Single	1	1
	Multiple	4.67 (2.98, 7.32)	5.48 (3.45, 8.71) ***
Place of delivery			
	Home	1	1
	Health facility	0.69 (0.49, 0.97)	0.68 (0.46, 0.99) *
Sex of baby			
	Male	1	1
	Female	0.62 (0.48, 0.80)	0.62 (0.48,0.81) ***
Apply anything on the umbilical cord after delivery			
	No	1	1
	Yes	0.66 (0.44, 0.99)	0.75 (0.47, 1.18)
Perceived size of baby			
	Small	1	1
	Medium	0.80 (0.57, 1.11)	0.80 (0.56, 1.13)
	Large	1.02 (0.70,1.51)	1.00 (0.64, 1.43)
Types of a high-risk fertility behavior			
	No any risk	1	1
	Mother age <18 years	1.34(.67, 2.67)	1.27 (0.63,2.52)
	Mother age > 34 years	1.16 (0.23, 5.84)	1.33 (0.25, 7.01)
	Birth interval <24 months	1.38 (1.00, 2.91)	1.52 (0.90, 2.58)
	Births order >3	0.84 (0.58, 1.23)	1.21 (0.79, 1.87)
	Double risks	1.44 (1.06, 1.96)	2.17 (1.52, 3.08) ***
	Triple risks	2.63 (1.30, 5.27)	3.69 (1.80, 7.55) ***

## Discussion

This study revealed that about 62.1%, 24%, and 2.3% of women were engaged at least in one, two, and three high-risk fertility behaviors, respectively, higher than the study done in nine East African countries (57.7% and 21.6% of women have experienced at least one and two high-risk fertility behaviors, respectively) [20], authors believed that, in Ethiopia, child marriage practice and unmet need for family planning are highly prevalent, about 58% and 22% of women are married before age of 18 years and unmet need for family planning, respectively [8]. Early marriage also affects the educational attainment of girls. Education helps them to discuss with partners and make joint decisions on family planning and family size. Furthermore, the positive effect of education on contraceptive use may be associated with the delay in marriage and first pregnancy [22, 23].

To the best of our knowledge, this study is the first to assess the impact that single and combinations of maternal high risk- fertility behaviors have on under-five mortality. Our finding shows that children born to women who engaged in two or more high-risk fertility behaviors were more likely to die before they celebrate their fifth birthday. Children born to women who engaged in double high-risk fertility behaviors were more likely to die as compared to children born to women who were no high-risk fertility behavior. This finding was supported by previous studies [16, 24]. Moreover, the odds of under-five mortality among children of women who were engaged in three high-risk fertility behaviors was higher compared to children of women who have not engaged in any high-risk fertility behavior [16].

Besides, this finding shows that the higher the number of high-risk fertility behaviors engaged by children’s mothers the higher the odds of under-five mortality. The odds of dying under-five children compared to that of children of women who have not engaged in any high-risk fertility behaviors with the number of odds from 2.17 for children born to women who were engaged in two high-risk fertility behaviors, and 3.69 for children born to women engaged in the three high-risk fertility behaviors. Previous studies suggested that exposure to multiple high-risk fertility behaviors predicted a higher level of depressive symptoms, adverse childbirth outcomes, a higher chance of receiving inappropriate preventive, and curative services, and an increase in the likelihood of inadequate child care practices [16].

Younger mothers are vulnerable to malnutrition and are not physically mature, which directly affects fetal development and results in low birth weight and infant deaths. Young mothers are also associated with less experienced in child care associated with child death [25–28]. Pregnancy among women aged above 34 years results in congenital and chromosomal abnormality due to hormonal disorder and low uteroplacental blood flow which increases fetal complications. And also it causes preterm birth with resultant infant death [29]. Short birth interval women cannot recover their nutritional stores which may results in malnutrition of the next pregnancy thereby increasing the risk of anemia, intrauterine growth retardation, and low birth weight [30, 31]. Short birth interval is also associated with placenta abruption, and premature rupture of the membrane may increase the risk of neonatal mortality [30]. However, the current study shows that single high-risk fertility behavior was not associated with under-five mortality. This finding was agreed with the previous study; too early childbearing (<18 years) [16], too late childbearing age [24], and too many births (>3) [16, 24] were not associated with under-five mortality. However, short birth interval space (<24 months) [16, 24] and too late childbearing age (>40) [16] were associated with under-five mortality. The reason might be the difference in socio-economical, health facilities accessibility, child care practice, and culture among participants that directly determine the survival of children [10–13].

Furthermore, other predictors of under-five death found in our study were being female children, children from large family sizes, children from households with improved toilets, and those born at health facilities were less likely to die, while multiple babies were more likely to die, which is supported by other previous findings [32–35]. Home delivery and unskilled attendants are one of the health services challenges in Ethiopia, only one-fourth of women gave birth at health facilities. Unskilled birth attendants do not have the skill to detect and manage complications arising during childbirth, thereby contributing to a high rate of under-five deaths. One of the primary causes of illness and death among children in low- and middle-income countries like Ethiopia is a lack of improved toilets. In Ethiopia, toilet coverage is inadequate; according to this report, only 10% of households have improved toilets. Multiple pregnancies can cause growth retardation and preterm, both of which are major risk factors for under-five mortality [35]. Furthermore, being twins may result in undernutrition due to insufficient breast milk, as well as infections due to incorrect formula feeding and cow’s milk feeding. The main strength of this study was that data come from a large nationally representative survey. Besides, our findings offer important insight into maternal high-risk fertility behaviors and their effect on under-five mortality in Ethiopia. However, this study might be subjected to recall bias, since EDHS was based on self-report.

## Conclusion

This study was analyzed the association of single and a combination of maternal high-risk fertility behaviors and under-five mortality in Ethiopia using EDHs 2016 secondary data. The current analysis shows that the presence of two or more combinations of maternal high-risk fertility behavior was increased the risk of under-five mortality. Hence, special emphasis should be given to children of women who engage in two or more high-risk fertility behaviors and multiple babies. Furthermore, enhancing toilet coverage and health facility deliveries may reduce child mortality. Increasing access to family planning services, preventing child marriage, and raising awareness on high-risk reproductive behaviors also should be strengthened.
